# The digital evolution of surgical planning: a systematic review of immersive and interactive technologies

**DOI:** 10.3389/fsurg.2026.1764132

**Published:** 2026-02-26

**Authors:** Waleed Daifallah Khubzan, Shuruq Awad Alharati, Rimas Warid Aljuaid, Dhay Saleem Algethami, Suhaiyh Sanad Alotibi, Haya Msfeir Alotaibi, Shaden Sultan Aljuaid, Rimas Salem Almalki, Mohammad Eid M. Mahfouz

**Affiliations:** 1 Faculty of Medicine, Taif University, Taif, Saudi Arabia; 2 Surgery Department, Faculty of Medicine, Taif University, Taif, Saudi Arabia

**Keywords:** augmented reality, immersive technology, intraoperative navigation, mixed reality, preoperative visualization, surgical planning, systematic review, virtual reality

## Abstract

**Background:**

Immersive and interactive technologies such as Virtual Reality (VR), Augmented Reality (AR), and Mixed Reality (MR) are reshaping surgical planning by enhancing anatomical visualization, enabling personalized procedures, and improving intraoperative navigation and decision-making across diverse surgical specialties.

**Methods:**

This systematic review was conducted in accordance with the PRISMA guidelines, and was registered in PROSPERO (CRD420251066149), analyzing 30 studies (1,270 participants) from PubMed, Google Scholar Web of Science and Ovid MEDLINE up to February 2025. Included studies evaluated VR, AR, or MR in preoperative or intraoperativesurgical planning, reporting outcomes on accuracy, time efficiency, or plan modifications. Risk of bias was assessed using RoB 2.0 for RCTs and ROBINS-I for non-randomized studies.

**Results:**

VR was the most utilized technology (17 studies), improving spatial understanding and prompting plan modifications in 32%–52% of cases (e.g., lung segmentectomies, TAVR). AR (8 studies) enhanced intraoperative accuracy, reducing pedicle screw placement errors (98% vs. 91.7% control) and procedure times (e.g., 50% faster spinal screw placement). MR (2 studies) demonstrated potential in reducing thoracic epidural needle adjustments (7.2 vs. 14.4 movements) and sentinel node biopsy durations (3.6 vs. 7.9 min). Heterogeneity in study designs and outcomes limited meta-analysis.

**Conclusion:**

VR enhanced anatomical understanding and preoperative planning, while AR, and MR were better for procedural accuracy and intraoperative workflow. Future multicenter trials with standardized protocols are needed to establish long-term clinical efficacy and cost-effectiveness in diverse surgical settings.

**Systematic Review Registration:**

https://www.crd.york.ac.uk/PROSPERO/view/CRD420251066149, PROSPERO CRD420251066149.

## 1 Introduction

Visualization of organs in 3-dimensional (3D) medical imaging has gained significance in medical care in the past decade, especially in the field of surgery. The use of 3D models helps to understand and estimate the size and shape of the internal organs of each patient before performing the surgical procedure. It allows surgeons to better prepare for surgery, determine the ideal surgical procedure tailored for each patient, and better prediction of the final surgery outcomes thus delivering better treatment to patients (1). This has been reinforced by advancements in extended reality applications in radiology, which have shown potential to enhance both diagnostic precision and preoperative planning (2).

Immersive and interactive technologies such as Virtual Reality (VR), Augmented Reality (AR), and Mixed Reality (MR) have emerged as transformative tools capable of bridging the gap between conventional imaging and intraoperative execution. VR enables fully immersive simulations by reconstructing three-dimensional anatomical models within a virtual environment, facilitating rehearsal of surgical approaches and improving spatial understanding (3). AR overlays virtual content onto the real-world operative field, allowing surgeons to integrate preoperative plans with live anatomy in real time (4). MR combines elements of both VR and AR, enabling dynamic interaction with holographic representations that can be manipulated and contextualized within the clinical workspace (5). Collectively, these modalities are redefining the possibilities of preoperative preparation and intraoperative navigation (6, 7).

Collectively, VR, AR, and MR have emerged as promising technologies to improve surgeons’ understanding of patient-specific anatomy, optimize operative strategies, and train in risk-free environments. For example, VR-based simulators have demonstrated efficacy in training laparoscopic and neurosurgical procedures, improving both technical skills and decision-making (8, 9). Similarly, AR has enabled surgeons to overlay critical anatomical information directly onto the patient during surgery, enhancing precision in orthopedic and maxillofacial procedures (1, 10). In cardiothoracic surgery, VR, AR, and MR applications are increasingly employed to refine operative strategies and optimize patient-specific planning (7). In neurovascular surgery, virtual stenting models and advanced simulation platforms have shown utility in planning interventions for cerebral aneurysms and complex vascular pathologies, providing improved spatial orientation and outcome prediction (11–13).

Several systematic reviews have examined individual modalities—such as VR for surgical training or AR for intraoperative navigation—but a comprehensive synthesis of immersive and interactive technologies across the spectrum of surgical planning is lacking. Prior work has primarily focused on subspecialty applications (e.g., thoracic or neurovascular planning) or imaging perspectives (e.g., radiology integration), without consolidating evidence across disciplines (2, 7). This review aims to bridge this gap by systematically evaluating the current evidence on the effectiveness of immersive and interactive technologies in surgical planning, with a focus on their impact on surgical accuracy, time efficiency, and decision-making.

## 2 Methods

### 2.1 Study design

This systematic review was conducted in accordance with the Preferred Reporting Items for Systematic Reviews and Meta-Analyses (PRISMA) guidelines. The protocol was registered on the International Prospective Register of Systematic Reviews (PROSPERO) with registration number (CRD420251066149).

### 2.2 Outcomes

The primary outcome was the accuracy of surgical planning and execution, assessed through measures such as anatomical localization and deviation errors. Secondary outcomes included surgical plan modifications, time efficiency (e.g., operative or planning time), radiation exposure, clinical outcomes (e.g., complication rates, blood loss), and user-related outcomes such as surgeon satisfaction and anatomical understanding. Outcomes were analyzed by technology type and surgical application phase (preoperative or intraoperative).

### 2.3 Study selection

Eligibility criteria were defined according to the PICOS (Population, Intervention, Comparator, Outcome, Study design) framework (14). Population (P) included patients, surgeons, or clinical teams involved in surgical planning. Intervention/Exposure (I) was the use of immersive or interactive technologies (virtual reality, augmented reality, mixed reality, or multi-modal platforms) applied to surgical planning. Comparators (C), where applicable, included conventional planning methods such as two-dimensional imaging, three-dimensional reconstructions, or standard visualization tools. Outcomes (O) included planning accuracy, spatial understanding, surgical decision-making, workflow efficiency, operative metrics, and user-centered outcomes. Study designs (S) included randomized controlled trials, prospective observational studies, feasibility studies, and retrospective analyses.

Studies were eligible for inclusion if they investigated or reported on the use of immersive technologies in any surgical specialty, including observational, feasibility, and comparative research. Studies were excluded if they were non-English publications or did not involve human surgical procedures, focused solely on educational or simulation purposes without clinical application, lacked outcome data, or were non-original publications such as reviews, editorials, conference abstracts, and technical notes.

### 2.4 Search strategy

A comprehensive and systematic literature search was conducted to identify studies that evaluate the effectiveness of immersive technologies for surgical planning before the operation. The databases searched included PubMed, Google Scholar, Web of Science and Ovid MEDLINE from inception until February 20, 2025. The search terms employed a combination of Medical Subject Headings (MeSH), and keywords related to (“Mixed reality” OR “Augmented reality” OR “Virtual reality”) AND (“Surgery” OR “Preoperative planning”). The electronic search strategy is provided in the (Supplementary Appendix A1). Titles and abstracts were independently screened by two reviewers, followed by full-text assessment by the same reviewers. Any disagreements were resolved through discussion and consensus, with consultation of a third reviewer when required. Formal inter-rater agreement statistics were not calculated.

The screening process has now been described in greater detail. Two independent reviewers screened titles and abstracts and subsequently assessed full-text articles for eligibility. Disagreements were resolved through discussion and consensus, with involvement of a third reviewer when necessary.

Formal inter-rater agreement statistics (e.g., Cohen's kappa) were not calculated, as this was not predefined in the study protocol and discrepancies were infrequent and resolved through consensus. This has now been explicitly stated in the Methods section for transparency. Embase, Scopus, and the Cochrane Library were not searched due to substantial overlap with MEDLINE- and Web of Science–indexed journals and institutional access limitations. Given the rapid technological evolution of immersive surgical planning, the selected databases were considered sufficient to capture the relevant peer-reviewed clinical literature.

### 2.5 Data extraction

Two independent reviewers extracted data from each included study using a standardized data collection form. Extracted information included study characteristics (author, year, country, sample size), type of immersive technology used (virtual reality, augmented reality, or mixed reality), imaging modality, 3D software platform, visualization hardware, surgical specialty, and phase of application (preoperative or intraoperative). Outcome data were also collected, including accuracy metrics, surgical plan changes, time efficiency measures, radiation exposure, clinical outcomes, and user-reported experiences.

### 2.6 Quality assessment

To evaluate the methodological quality and risk of bias of the included studies, two validated tools were employed based on the study design. RoB 2.0 Tool (Revised Cochrane risk-of-bias tool for randomized trials) was used for assessing randomized controlled trials (RCTs). This tool evaluates five domains: bias arising from the randomization process, deviations from intended interventions, missing outcome data, measurement of the outcome, and selection of the reported result. Each domain is rated as having low, some concerns, or high risk of bias (15).

ROBINS-I Tool (Risk Of Bias In Non-randomized Studies of Interventions) was used to assess prospective observational and feasibility studies. This tool examines seven domains of bias: due to confounding, selection of participants, classification of interventions, deviations from intended interventions, missing data, measurement of outcomes, and selection of reported results. It is suitable for evaluating non-randomized studies that aim to estimate the effects of interventions, providing an overall risk of bias rating as low, moderate, serious, or critical (16). All included studies were independently assessed by two reviewers using the appropriate tool for their design. Any disagreements were resolved through discussion to ensure consistent and accurate judgments.

### 2.7 Data synthesis

Extracted data were synthesized narratively due to the heterogeneity of study designs, technologies, procedures, and outcome measures. Studies were grouped based on the type of immersive technology used (virtual, augmented, or mixed reality) and the phase of surgical application (preoperative or intraoperative). Key outcomes such as accuracy, surgical plan modification, time efficiency, radiation exposure, clinical results, and user feedback were summarized and compared across studies. Quantitative findings were tabulated to highlight trends, but no meta-analysis was performed due to variability in outcome reporting and measurement methods.

## 3 Results

### 3.1 Search results

**Figure 1:**
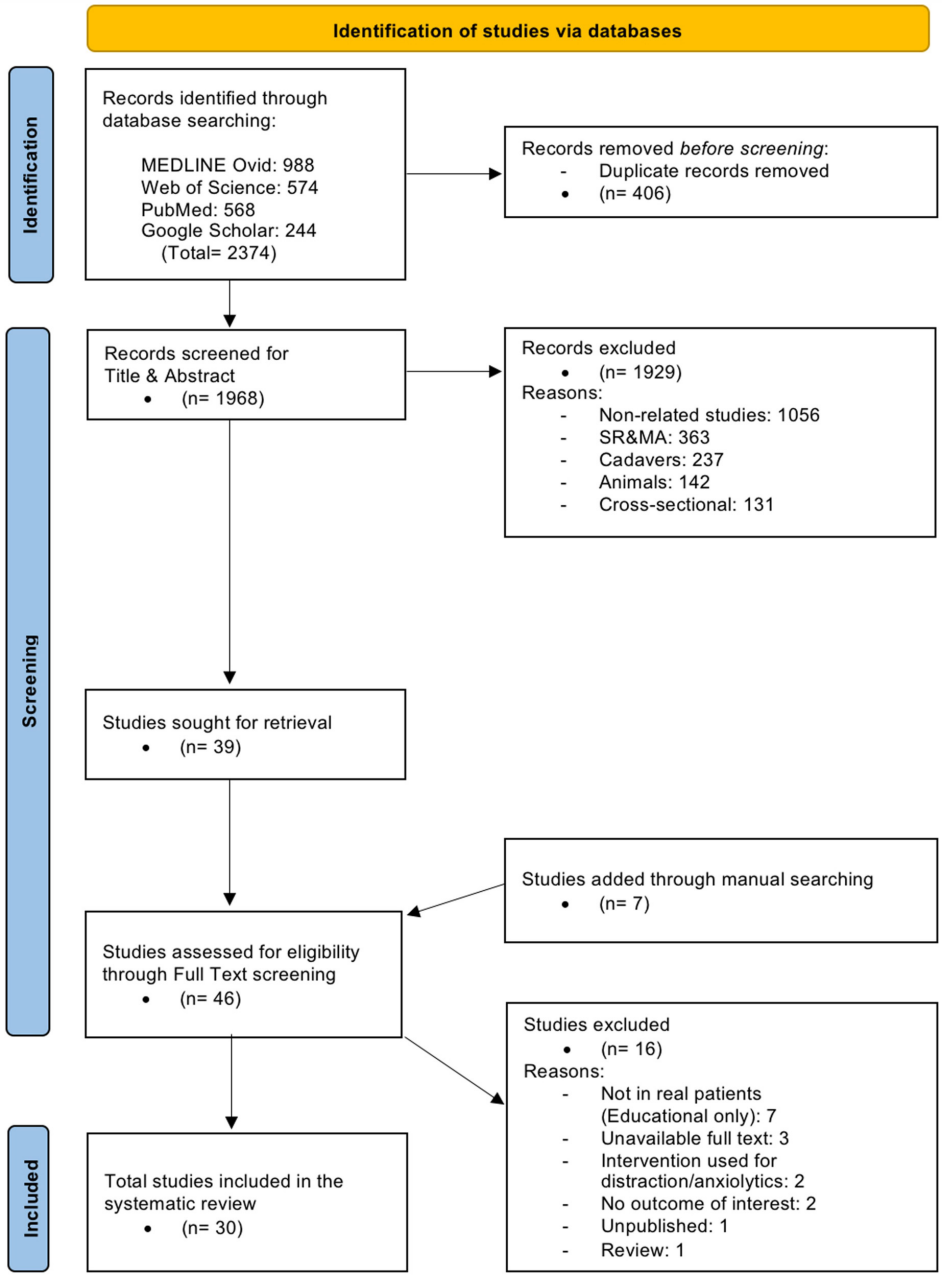
PRISMA Flow Diagram of Study Selection. PRISMA flow diagram illustrating the study selection process, including database identification, duplicate removal, title and abstract screening, full-text eligibility assessment, and final inclusion of studies in the systematic review.

The initial search across four electronic databases (PubMed, Google Scholar, Web of Science and Ovid MEDLINE) yielded a total of 2,374 records (988 from MEDLINE Ovid, 574 from Web of Science, 568 from PubMed, and 244 from Google Scholar) (Figure 1). After removal of 406 duplicate records, 1,968 studies remained for title and abstract screening. Of these, 1,929 studies were excluded for the following reasons: non-related studies (*n* = 1,056), systematic reviews or meta-analyses (*n* = 363), cadaveric studies (*n* = 237), animal studies (*n* = 142), and cross-sectional studies (*n* = 131).

A total of 39 studies were sought for full-text retrieval. Through manual searching, we identified an additional seven studies, bringing the total number of articles subjected to full-text screening to 46. Manual searching included two strategies: First one is backward citation searching of relevant systematic reviews to identify original studies that might have been missed during database searches. While the second one is snowballing (pearl growing), in which the “related articles” and “similar studies” functions in PubMed, Scopus, and Google Scholar were used when retrieving known eligible articles.

Following full-text review, 16 studies were excluded (educational-only interventions without real patients, *n* = 7 (4, 17–22); unavailable full text, *n* = 3 (23–25); interventions designed solely for distraction or anxiolysis, *n* = 2 (26, 27); no relevant outcomes, *n* = 2 (28, 29); unpublished, *n* = 1 (30); review article, *n* = 1 (31)). Ultimately, 30 studies met the inclusion criteria and were included in this systematic review (7, 32–60).

### 3.2 Study & participants characteristics

A total of 30 studies were included in this systematic review, encompassing 1,270 participants, including both patients and clinicians (Table 1). The most common study design was the randomized controlled trial, accounting for 14 studies. This was followed by 9 prospective observational studies, 3 prospective feasibility studies, 3 retrospective studies (including case series and observational), and 1 preclinical comparative study. Participants were recruited across a wide geographic distribution, with studies conducted in China, USA, Germany, France, Netherlands, and other countries.

**Table 1:** Characteristics of the included studies.

Author (Year)	Design	Location	Sample size	Technology	Procedure
Qu et al. (2015)	Randomized Controlled Trial	China	20	AR	Mandibular Distraction Osteogenesis
Diana et al. (2017)	Prospective Non-Randomized	France	58	VR, AR	Robotic cholecystectomy
Sampogna et al. (2017)	Prospective Feasibility Study	Italy	15	VR	Pancreatic, hepatic, renal surgeries
Hummelink et al. (2018)	Randomized Controlled Trial	Netherlands	60	AR	Breast reconstruction
Auloge et al. (2018)	Randomized Controlled Trial	France	20	AR	Vertebroplasty
Zheng et al. (2018)	Randomized Controlled Trial	China	30	VR	PELD
Pereira et al. (2019)	Prospective Observational	Chile	30	AR	SCIP flap surgery
Shirk et al. (2019)	Randomized Controlled Trial	United States	92	VR	RAPN
Wei et al. (2019)	Randomized Controlled Trial	China	40	MR	Kyphoplasty
Samer Alsofy et al. (2020)	Retrospective observational study	Germany	30	VR	Skull base meningioma
Alsofy et al. (2020)	Retrospective observational study	Germany	26	VR	ACoA aneurysm clipping
Louis et al. (2020)	Retrospective case series	USA	49	VR, AR, XR	Craniotomy, aneurysm clipping
Sugiyama et al. (2020)	Prospective Observational	Japan	38	VR	Neurosurgery
D'Urso et al. (2020)	Prospective Feasibility Study	France	27	FLER+NIR camera	Colorectal surgery
Abjigitova et al. (2021)	Prospective Observational	Netherlands	6	VR	Ascending aortic surgery
Beheiry et al. (2021)	Prospective Randomized	France	25	VR	Breast tumor localization
Huettl et al. (2021)	Preclinical Comparative	Germany	30 clinicians	VR	Liver surgery
McDonald et al. (2021)	Prospective Observational	USA	15	VR	RAPN
Shakya et al. (2021)	Randomized Controlled Trial	China	50	VR	Mandibular condylar fracture fixation
Sadeghi et al. (2021)	Prospective Observational	Netherlands	10	VR	VATS
Roethe et al. (2021)	Randomized Controlled Trial	Germany	55	AR	Neurosurgery
Staubli et al. (2022)	Randomized Controlled Trial	Switzerland	74 clinicians	VR	Cholecystectomy
Ruyra et al. (2022)	Prospective	Spain	11	VR	TAVR
Shirk et al. (2022)	Randomized Controlled Trial	USA	92	VR	RALP
Bakhuis et al. (2023)	Prospective Observational	Netherlands	50	VR	Lung segmentectomy
Lee et al. (2023)	Randomized Controlled Trial	South Korea	20	AR	Breast tumor localization
Saruwatari wt al. (2023)	Randomized Controlled Trial	USA	34	AR	Ultrasound-guided vascular access
Weigelmann et al. (2023)	Randomized Controlled Trial	Canada	83	MR	Thoracic epidural placement
Colcuc et al. (2024)	Prospective Observational	Germany	30	VR	Tibial plateau fracture
Ma et al. (2024)	Randomized Controlled Trial	China	150	AR	Spinal pedicle screw placement

VR, virtual reality; AR, augmented reality; MR, mixed reality; XR, extended reality; FLER, fluorescence-based enhanced real; NIR, near-infrared; RAPN, robotic assisted partial nephrectomy; VATS, video-assisted thoracoscopic segmentectomy; TAVR, transcatheter aortic valve replacement; RALP, robotic-assisted laparoscopic radical prostatectomy; ACoA, anterior communicating artery; SCIP, superficial circumflex iliac artery perforator; PELD, percutaneous endoscopic lumbar discectomy.

Virtual Reality was the most commonly used technology, applied in 17 studies. Augmented Reality was used in 8 studies, Mixed Reality in 2 studies. Multi-modalities, and other immersive tools such as Extended Reality and FLER (Fluorescence-based enhanced real) combined with NIR (Near-infrared) cameras appeared in isolated cases.

The included procedures covered a diverse range of surgical specialties. Commonly studied interventions included neurosurgery, breast tumor localization, robotic-assisted urological surgeries (e.g., RAPN and RALP), spinal surgery, cholecystectomy, and vascular access techniques. Other notable areas included colorectal, hepatic, orthopedic, thoracic, and cardiovascular surgeries.

### 3.3 Characteristics of the used technologies

Across the 30 included studies, a range of immersive and interactive technologies were employed, primarily VR, AR, and MR, often integrated with preoperative imaging modalities such as CT, MRI, ultrasound, and angiographic datasets (Table 2). Most studies leveraged these technologies for preoperative planning (*n* = 23), with several extending applications to intraoperative navigation or guidance (*n* = 14), and a few specifically targeting tumor localization or vascular access. Intraoperative applications often relied on real-time overlays and holographic projections, while preoperative uses focused on anatomical visualization, surgical rehearsal, and spatial orientation.

**Table 2:** Characteristics of the used interventions.

Author (Year)	Technology used	3D software/platform	Visualization hardware	Application purpose
Qu et al. (2015)	AR + CT	Materialise Mimics	—	Preoperative & intraoperative navigation
Diana et al. (2017)	VR/AR + MRCP	Visible Patient suite	—	Preoperative & intraoperative navigation
Sampogna et al. (2017)	VR + CT/MRI	3D Slicer	Oculus Rift (HMD)	Preoperative & intraoperative navigation
Hummelink et al. (2018)	AR + CTA	Custom-built AR projection system	—	Preoperative & intraoperative navigation
Auloge et al. (2018)	AR + C-arm fluoroscopy	—	—	Intraoperative navigation
Zheng et al. (2018)	VR + CT Spiral	M-Visioneer	—	Preoperative navigation
Pereira et al. (2019)	AR + CTA	AVA	—	Preoperative & intraoperative navigation
Shirk et al. (2019)	VR + CT/MRI	Reveal software (Ceevra)	—	Preoperative planning
Wei et al. (2019)	MR + CT	CAD+M3D digital medical software	HoloLens	Preoperative & intraoperative navigation
Samer Alsofy et al. (2020)	VR + MRI/CTA	3D Slicer	HMD	Preoperative navigation
Alsofy et al. (2020)	VR + MRA/CTA	3D Slicer	HMD	Preoperative navigation
Louis et al. (2020)	VR/AR/XR + CT/MRI/Angiography	—	—	Preoperative planning & intraoperative navigation
Sugiyama et al. (2020)	VR + CT/MRI	BananaVision	HMD	Preoperative planning
D’Urso et al. (2020)	FLER	—	—	Intraoperative perfusion evaluation
Abjigitova et al. (2021)	VR + CT	3D Slicer	Oculus Rift (HMD)	Preoperative planning
Beheiry et al. (2021)	VR + MRI	DIVA software	HTC Vive headset	Preoperative tumor localization
Huettl et al. (2021)	VR + CT	Synapse 3D (Fujifilm)	HMD	Preoperative planning
McDonald et al. (2021)	VR + CT/MRI	Ceevra	HMD	Preoperative planning
Shakya et al. (2021)	VR + CT	Materialise Mimics	HMD	Preoperative planning
Sadeghi et al. (2021)	VR + CT	LungQ (Thirona)	PulmoVR, EVOCS (Thirona)	Preoperative planning
Roethe et al. (2021)	AR + MRI/DTI, nTMS	—	HMD	Intraoperative navigation
Staubli et al. (2022)	VR + MRCP	Materialise Mimics	SpectoVR (HMD)	Preoperative & intraoperative navigation
Ruyra et al. (2022)	VR + MDCT	3D Heart Navigator	HMD	Preoperative planning
Shirk et al. (2022)	VR + MRI	Reveal software (Ceevra)	HMD	Preoperative planning
Bakhuis et al. (2023)	VR + CT	Pulmo3D	PulmoVR, MedicalVR (HMD)	Preoperative planning
Lee et al. (2023)	AR + CT/MRI/Ultrasound/Mammography	SKIA Processor	—	Preoperative localization
Saruwatari et al. (2023)	AR + Ultrasound	SolidWorks	HoloLens	Intraoperative vascular access
Weigelmann et al. (2023)	MR + Ultrasound	—	HoloLens	Intraoperative guidance
Colcuc et al. (2024)	VR + CT	OsiriX	HMD	Preoperative planning
Ma et al. (2024)	AR + CT	—	HoloLens	Intraoperative guidance

VR, virtual reality; AR, augmented reality; MR, mixed reality; XR, extended reality; FLER, fluorescence-based enhanced real; NIR, near-infrared; 3D, 3-dimensional; CT, computer tomography; MRI, magnetic resonance imaging; MRCP, magnetic resonance cholangiopancreaticography; CTA, computer tomography angiography; MRA, magnetic resonance angiography; DTA, diffusion tensor imaging; nTMS, navigated transcranial magnetic stimulation; MDCT, multidetector computer tomography; AVA, advanced vessel analysis; CAD, computer-aided design; HMD, head mounted display.

A variety of 3D software platforms supported model reconstruction and visualization. Frequently used platforms included 3D Slicer, Materialise Mimics, Reveal (Ceevra), OsiriX, and vendor-specific programs such as Pulmo3D, Synapse 3D, and BananaVision. In studies using AR or MR, Microsoft HoloLens was the most common hardware, while VR studies typically employed head-mounted displays (HMDs) such as the Oculus Rift, HTC Vive, and SpectoVR.

Notably, the immersive technologies were tailored to the specific surgical domain and task. Neurosurgical and thoracic procedures often utilized MR and AR for real-time intraoperative support, while urologic, hepatobiliary, and orthopedic applications favored VR-based planning.

### 3.4 Quality assessment results

Among the 16 RCTs assessed using RoB 2, 10 studies raised to have some concerns, mainly due to the first (D1) and second (D2) domains. Four studies were judged to have an overall low risk of bias, while only 2 studies had an overall high risk of bias, primarily due to the first and second domains once again. No study was rated as high risk overall. The domains of missing outcome data (D3) and reported results selection (D4) were generally well-addressed across trials.

The risk of bias across the remaining 14 included studies was assessed using the ROBINS-I tool. Most studies exhibited significant methodological limitations, with 9 of the 14 studies rated as having a serious overall risk of bias and 4 studies judged to have critical risk of bias. Critical and serious risks were most frequently observed in the “bias due to confounding” domain. Bias in the selection of participants was primarily rated as moderate (9 studies), while the third (D3), fourth (D4), and fifth (D5) domains were predominantly judged as low risk for almost all of the studies.

Outcomes related to spatial understanding and preoperative planning accuracy were most frequently evaluated in randomized controlled trials, whereas workflow efficiency, feasibility, and user experience outcomes were predominantly reported in non-randomized studies. These differences were considered when interpreting the strength of evidence across outcomes. Finally, while the randomized studies demonstrated generally solid internal validity, the non-randomized evidence carried higher susceptibility to bias. These assessments highlight the need for cautious interpretation of observational findings and support the strength of conclusions drawn primarily from the randomized trials (Supplementary Appendix A2).

### 3.5 Accuracy outcomes

Out of the 30 included studies, 17 [9 RCTs (53%) and 8 observational/feasibility studies (47%)] reported quantitative or qualitative outcomes related to accuracy in surgical planning and execution as demonstrated in Table 3A. A wide range of procedures were evaluated, including cranial, spinal, maxillofacial, breast, hepatobiliary, thoracic, and orthopedic surgeries.

**Table 3A:** Accuracy outcomes of immersive technologies in surgical planning.

Author (Year)	Sample size	Technology	Procedure	Reported accuracy
Qu et al. (2015)	20	AR	Mandibular Distraction Osteogenesis	Osteotomy positioning was significantly better in the AR group (*p* < 0.01).
Diana et al. (2017)	58	VR, AR	Robotic cholecystectomy	VR identified 12 anatomical variants; 7 confirmed radiologically (*p* = 0.0037). CD-CBD junction visualized in 100% with VR, vs. 98.2% (NIR-C) and 96.2% (IOC).
Hummelink et al. (2018)	60	AR	Breast reconstruction	Better perforator identification with AR (62% vs. 41%, *p* = 0.02).
Auloge et al. (2018)	20	AR	Percutaneous vertebroplasty	Trocar placement accurate to 1.68 ± 0.25 mm (skin entry) and 1.02 ± 0.26 mm (tip); 100% feasibility.
Zheng et al. (2018)	30	VR	PELD	VR can predict a surgery-related angle and distance accurately except for depth.
Pereira et al. (2019)	30	AR	SCIP flaps	AR planning with ARM-PS was 100% accurate compared to handheld Doppler and intraoperative findings.
Louis et al. (2020)	49	VR, AR, XR	Craniotomy, aneurysm clipping	Improved anatomical guidance and craniotomy precision; gross total resection achieved in 85% (34/40).
D’Urso et al. (2020)	27	FLER + NIR camera	Colorectal surgery	FLER correlated with perfusion markers (rho = 0.76 and 0.71); better ischemic zone detection than unquantified fluorescence (*p* < 0.05).
Beheiry et al. (2021)	25	VR	Breast tumor localization	The accuracy of quadrant determination was significantly improved with VR for practicing surgeons (*P* = .01).
Shakya et al. (2021)	50	VR	Mandibular condylar fracture fixation	Deviation reduced: 0.639 mm (VR) vs. 0.995 mm (control), *p* < 0.001.
Sadeghi et al. (2021)	10	VR	VATS	100% intraoperative correlation with VR model.
Staubli et al. (2022)	74	VR	Cholecystectomy	Improved anatomical recognition and MRCP performance with VR (*p* < 0.05).
Bakhuis et al. (2023)	50	VR	Lung segmentectomy	Radical resection confirmed in 98% (49/50).
Lee et l. (2023)	20	AR	Breast tumor localization	AR localization was as effective as ultrasound-guided localization.
Weigelmann et al. (2023)	83	MR	Thoracic epidural placement	Reduced needle movements (7.2 vs. 14.4, *p* = 0.01).
Colcuc et al. (2024)	30	VR	Tibial plateau fracture classification	VR led to higher interrater reliability and improved classification confidence.
Ma et al. (2024)	150	AR	Spinal pedicle screw placement	98% of screws rated “excellent” in AR vs. 91.7% in control (*p* < 0.0003).

VR, virtual reality; AR, augmented reality; MR, mixed reality; FLER, fluorescence-based enhanced real; NIR, near-infrared; 3D, 3-dimensional; PR, printed models; SCIP, superficial circumflex iliac artery perforator; VATS, video-assisted thoracoscopic segmentectomy; PELD, percutaneous endoscopic lumbar discectomy; RAPN, robotic-assisted partial nephrectomy; ARM-PS, augmented reality for microsurgical planning with a smartphone; CD, cystic duct; CBD, common bile duct; MRCP, magnetic resonance cholangiopancreatography.

Augmented Reality demonstrated consistent improvements in surgical accuracy across several domains. Notable findings include superior osteotomy positioning in mandibular distraction (*p* < 0.01), enhanced perforator identification in breast reconstruction (62% vs. 41%, *p* = 0.02), and improved pedicle screw placement accuracy in spine surgery, with 98% of screws rated as “excellent” compared to 91.7% in the control group (*p* < 0.0003). AR was also shown to be equally effective as ultrasound for breast tumor localization, and it enabled accurate preoperative planning in microsurgical flap procedures and spinal instrumentation.

Virtual Reality was similarly effective in enhancing spatial understanding and reducing deviation. For instance, VR significantly reduced deviation in mandibular condylar fracture fixation (0.639 mm vs. 0.995 mm, *p* < 0.001), improved quadrant accuracy for breast tumor localization in experienced surgeons (*p* = 0.01), and achieved 100% intraoperative correlation in thoracoscopic segmentectomy. In robotic cholecystectomy, VR facilitated improved biliary anatomy identification, recognizing anatomical variants with a high confirmation rate (*p* = 0.0037), and it improved MRCP interpretation in another cholecystectomy study (*p* < 0.05).

Mixed Reality also showed benefits, particularly in reducing needle adjustments during thoracic epidural placement (7.2 vs. 14.4 movements, *p* = 0.01). One study using FLER found significant correlation with perfusion markers, enabling better intraoperative ischemia detection (*p* < 0.05).

Across the 17 studies reporting accuracy outcomes, 16 (94%) demonstrated a favorable directional effect for immersive technologies compared with conventional approaches. Quantitative improvements included reduced deviation, higher placement grades, and improved localization success, with reported absolute differences ranging from 6% to 21% depending on procedure. No study reported inferior accuracy with immersive technology, supporting a consistent directional benefit despite heterogeneity in outcome metrics.

### 3.6 Time efficiency outcomes

A total of 16 [8 RCTs (50%) and 8 observational/feasibility studies (50%)] studies reported on the impact of immersive technologies on time efficiency, primarily focusing on reduced operative times, faster imaging interpretation, and improved procedural workflows (Table 3B). These studies involved various surgical specialties, including hepatobiliary, orthopedic, neurosurgical, vascular, and reconstructive procedures.

**Table 3B:** Time efficiency improvements after use of immersive technologies.

Author (Year)	Sample size	Technology	Procedure	Time efficiency outcome
Diana et al. (2017)	58	VR + AR	Robotic cholecystectomy	Time to obtain 1mages shorter with NIR-C than AR (*p* = 0.008); 5R-AR slower but had higher 1mage quality.
Sampogna et al. (2017)	15	VR	Pancreatic, hepatic, renal surgeries	VR enabled more efficient 3D modeling and planning workflows.
Hummelink et al. (2018)	60	AR	Breast reconstruction	Flap harvest time reduced by 19 min (136 ± 7 5 s. 155 ± 7, *p* = 0.012); pre-op mapping time reduced (2.3 5 s. 20 min, *p* < 0.001).
Auloge et al. (2018)	20	AR	Vertebroplasty	Fluoroscopy time reduced to 5.2 s 5 s. 10.4 s (*p* = 0.005).
Zheng et al. (2018)	30	VR	PELD	Fluoroscopy and location times significantly reduced (*p* < 0.001).
Pereira et al. (2019)	30	AR	SCIP flap surgery	Flap harvest time reduced by 20% (72 5 s. 90 min).
Shirk et al. (2019)	92	VR	RAPN	Clamp time shorter 1n 5R group (not statistically significant).
Wei et al. (2019)	40	MR	Kyphoplasty	Operation and fluoroscopy times both significantly reduced (*p* < 0.05).
D’Urso et al. (2020)	27	FLER + NIR camera	Colorectal surgery	Average FLER analysis time: 7.8 min.
Beheiry et al. (2021)	25	VR	Breast tumor localization	MRI analysis time significantly lower with 5R (*p* < 0.001).
Huettl et al. (2021)	30	VR	Liver surgery	Tumor assignment time significantly shorter with 3D PR than PDF or 5R (*p* < 0.001).
Shakya et al. (2021)	50	VR	Mandibular condylar fracture fixation	Operation time: 180.6 min (VR) 5 s. 223.2 min (control).
Saruwatari et al. (2023)	34	AR	Ultrasound-guided 5ascular access	Completion time: 11.5 5 s. 18.5 s (*p* = 0.04).
Weigelmann et al. (2023)	83	MR	Thoracic epidural placement	Procedure time: 4.5 5 s. 7.3 min (*p* = 0.02).
Colcuc et al. (2024)	30	VR	Tibial plateau fracture	Operative time reduced: 156 5 s. 172 min (*p* < 0.001).
Ma et al. (2024)	150	AR	Spinal pedicle screw placement	Screw placement time reduced: 16.3 ± 9.9 min 5s. 30.3 ± 10.4 min (*p* < 0.05); fluoroscopy time also reduced (*p* < 0.05).

VR, Virtual Reality; AR, Augmented Reality; MR, Mixed Reality; FLER, Fluorescence-based enhanced real; NIR, Near-infrared; 3D, 3-Dimensional; PR, Printed models; SCIP, Superficial circumflex iliac artery perforator; VATS, Video-assisted thoracoscopic segmentectomy; PELD, Percutaneous endoscopic lumbar discectomy; RAPN, Robotic-assisted partial nephrectomy.

Augmented Reality was associated with consistent time-saving benefits in intraoperative and preoperative contexts. For example, AR significantly reduced flap harvest time in breast reconstruction (136 ± 7 vs. 155 ± 7 min, *p* = 0.012) and mapping time (2.3 vs. 20 min, *p* < 0.001). In spinal surgery, AR decreased pedicle screw placement time by nearly 50% (*p* < 0.05), and in ultrasound-guided vascular access, AR reduced the completion time from 18.5 to 11.5 s (*p* = 0.04). Similarly, vertebroplasty procedures showed reduced fluoroscopy time with AR (*p* = 0.005).

Virtual Reality contributed to streamlined planning and faster execution in several procedures. VR reduced operative time in mandibular fracture fixation (180.6 vs. 223.2 min), MRI interpretation time in breast tumor localization (*p* < 0.001), and operative time in tibial plateau fracture management (156 vs. 172 min, *p* < 0.001). However, some studies reported non-significant time gains, such as clamp time reduction in robotic-assisted nephrectomy.

Mixed Reality also demonstrated efficiency gains. In thoracic epidural placement, MR shortened the procedure duration (4.5 vs. 7.3 min, *p* = 0.02). Additionally, technologies like FLER offered rapid perfusion analysis with an average processing time of 7.8 min, contributing to real-time intraoperative decision-making.

Among the 16 studies assessing efficiency, 13 (81%) reported measurable reductions in time-related outcomes, including operative time, planning time, or fluoroscopy exposure. Absolute time savings ranged from seconds (6–9 s for vascular access) to minutes (3–19 min for operative or procedural duration), and up to ∼50% reductions in specific tasks such as pedicle screw placement time. However, 3 studies reported non-significant or mixed effects, indicating that efficiency gains were context-dependent and less consistent than accuracy outcomes.

### 3.7 Surgical plan modifications

A total of 12 [2 RCTs (17%) and 10 observational/feasibility studies (83%)] studies evaluated the impact of immersive technologies—primarily Virtual Reality—on surgical plan modifications (Table 3C). These studies consistently reported changes in preoperative strategies, anatomical assessments, and intraoperative decision-making across various surgical specialties, including neurosurgery, urology, cardiothoracic, and vascular interventions.

**Table 3C:** Surgical plan modifications following the use of immersive technologies.

Author (Year)	Sample size	Technology	Procedure	Plan Modification Summary
Diana et al. (2017)	58	VR + AR	Robotic cholecystectomy	VR planning changed the surgical approach in some cases.
Sampogna et al. (2017)	15	VR	Pancreatic, hepatic, and renal surgeries	Surgical strategy was altered in some laparoscopic nephrectomy procedures after 3D reconstruction.
Samer Alsofy et al. (2020)	30	VR	Skull base meningioma	VR influenced recommended head positioning and surgical approach significantly (*p* < 0.03).
Alsofy et al. (2020)	26	VR	ACoA aneurysm clipping	VR altered the recommended head position and approach (*p* < 0.005).
Sugiyama et al. (2020)	38	VR	Neurosurgery	VR enhanced decision-making in 61.1% and understanding in 83.3% of cases.
Abjigitova et al. (2021)	6	VR	Ascending aortic surgery	Pre-op plan adjusted in 33% (2/6) of cases based on 3D-VR evaluation.
Huettl et al. (2021)	30	VR	Liver surgery	90% of users felt VR positively influenced planning, and enhanced decision-making.
McDonald et al. (2021)	15	VR	RAPN	In 6 out of 15 cases, surgical plans were modified after VR review.
Sadeghi et al. (2021)	10	VR	VATS	40% of cases had a change in target segment selection.
Ruyra et al. (2022)	11	VR	TAVR	5 of 11 cases (45.4%) had changes in approach, implantation depth, or access route due to VR insights.
Shirk et al. (2022)	92	VR	RALP	32% of surgeons changed their plan after VR model.
Bakhuis et al. (2023)	50	VR	Lung segmentectomy	Surgical plan altered in 52% of cases; confirmed by pathology in 98% (49/50).

VR, Virtual Reality; AR, Augmented Reality; 3D, 3-Dimensional; ACoA, Anterior communicating artery; RAPN, Robotic assisted partial nephrectomy; VATS, Video-assisted thoracoscopic segmentectomy; TAVR, Transcatheter aortic valve replacement; RALP, Robotic-assisted laparoscopic radical prostatectomy.

VR-based planning frequently led to meaningful alterations in surgical strategies. In skull base and cerebrovascular procedures, VR influenced the choice of head positioning and surgical approach, with statistical significance reported in both skull base meningioma (*p* < 0.03) and anterior communicating artery aneurysm cases (*p* < 0.005). Similarly, in pancreatic, hepatic, and renal surgeries, 3D-VR modeling prompted changes in laparoscopic nephrectomy strategies.

VR applications also impacted planning in thoracic and vascular interventions. For example, lung segmentectomy plans were modified in 52% of cases, with accuracy confirmed by pathology in 98% of those revisions. In TAVR procedures, VR insights resulted in alterations in implantation depth, access route, or overall approach in 45.4% of cases. Similarly, target segment selection changed in 40% of video-assisted thoracoscopic surgeries (VATS).

Surgical decision-making also benefitted in robotic-assisted urologic surgeries. Among RALP cases, 32% of surgeons revised their preoperative plans after reviewing VR models. Likewise, 6 out of 15 surgeons modified their approach in robotic-assisted partial nephrectomy following VR review. Surgeons also reported enhanced anatomical understanding and preoperative confidence, with one study noting that 61.1% of decisions were improved and 83.3% of surgeons gained better comprehension through VR visualization.

Across the 12 studies evaluating decision impact, immersive technologies resulted in surgical plan modifications in approximately 30%–52% of cases, with reported rates ranging from 33% to 90% depending on procedure and assessment method. When explicitly quantified, absolute modification frequencies included 6/15 cases (40%), 5/11 cases (45.4%), and 2/6 cases (33%). These findings indicate a meaningful influence on surgical decision-making, although estimates varied widely and were predominantly derived from non-randomized studies.

### 3.8 Comparative synthesis of findings across technologies

Across the included studies, several overarching patterns emerged. Virtual Reality was most consistently associated with enhanced spatial understanding, particularly in preoperative planning, where it facilitated accurate anatomical visualization, improved surgical confidence, and often led to modifications in surgical strategies. Augmented Reality, in contrast, demonstrated its greatest strength in intraoperative applications, where real-time overlays contributed to improved surgical accuracy and substantial reductions in operative time. Mixed Reality, although less extensively studied, showed promising benefits in niche applications such as epidural placement, where it reduced both error rates and procedural duration. Despite these common trends, divergences were noted: while some VR-based applications demonstrated significant operative time reductions, others showed nonsignificant or modest effects, highlighting heterogeneity across specialties and procedures. Similarly, AR's intraoperative precision gains were consistently observed, but its added value in preoperative planning was less clear. Collectively, these findings suggest a complementary role of the different immersive technologies—VR strengthening preoperative planning, AR enhancing intraoperative execution, and MR offering targeted benefits in emerging clinical scenarios.

## 4 Discussion

**Figure 2:**
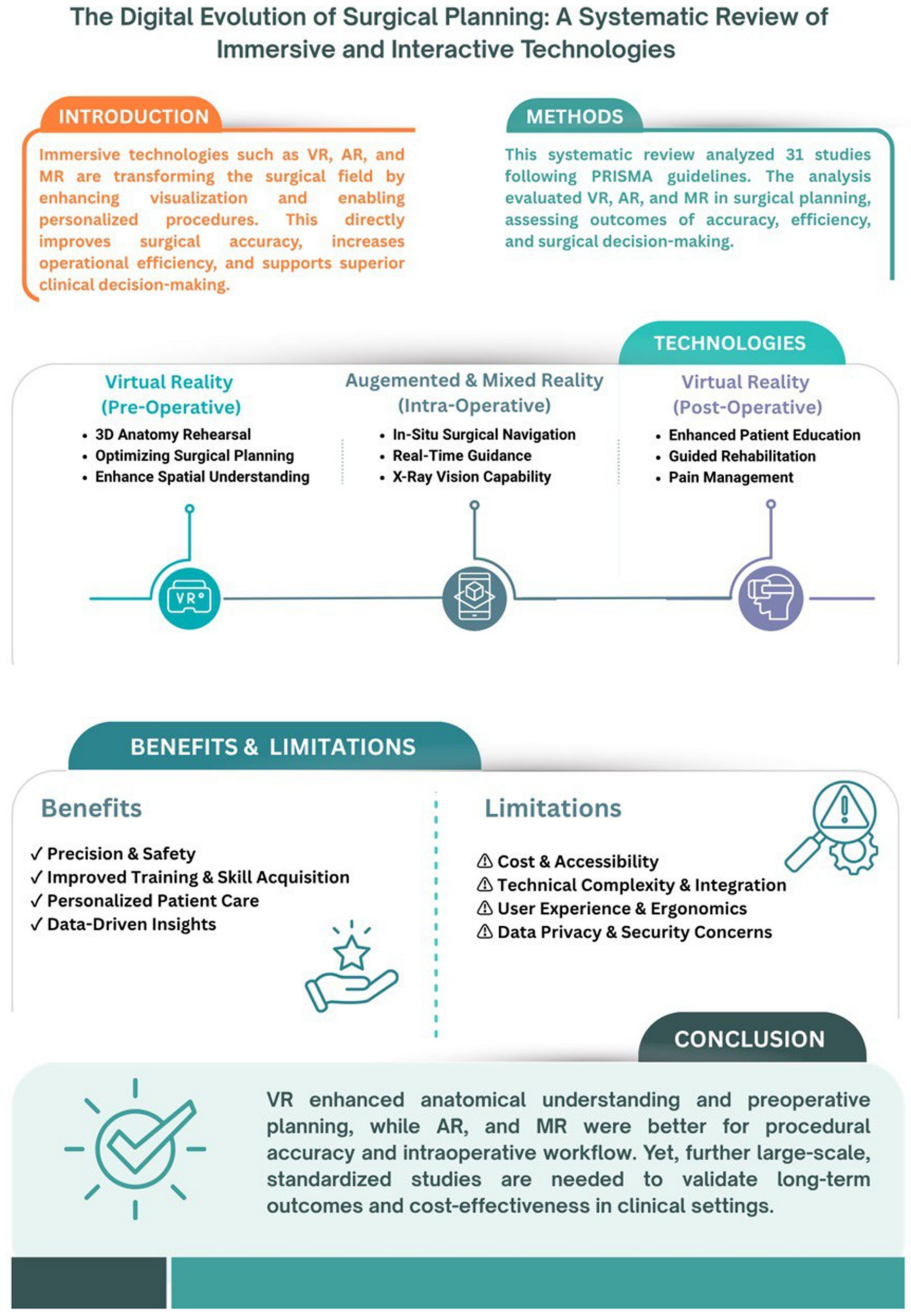
Conceptual Framework of Immersive and Interactive Technologies in Surgical Planning. Conceptual framework illustrating the role of immersive and interactive technologies in surgical planning, including Virtual Reality (VR), Augmented Reality (AR), and Mixed Reality (MR). The figure highlights their applications in preoperative planning, surgical simulation, intraoperative guidance, and education, along with reported outcomes such as enhanced anatomical visualization, improved surgical accuracy, workflow efficiency, and clinical decision-making support.

This systematic review focuses on investigating the application of immersive and interactive technologies such as Virtual Reality, Augmented Reality, Mixed Reality, and other extended reality technologies in the planning of surgical procedures and intraoperative workflows. Moreover, it addresses the impact of these technologies on precision, time efficiency, adjustment of preoperative plans across numerous surgical subspecialties. A visual summary is demonstrated in (Figure 2).

Our results provide evidence that supports the claim of the effectiveness of advanced immersive technologies in modern surgical planning. From the 30 studies included in our review, we found that VR was the most used modality with AR and MR following behind. Across these modalities, immersive tools consistently enhanced spatial understanding, procedural accuracy, intraoperative navigation, and surgeon confidence. While AR proved to be effective for real-time navigation in an imaging interventional scan during surgery VR substantially improved visualization and prompting major alterations to plans prior to operations. Despite the lack of extensive use, MR has shown encouraging results in certain thoracic surgeries before and during procedures. Importantly, the technologies collectively improved accuracy, shortened procedure times, and often led to revisions in surgical strategy, highlighting their growing value in precision-based surgery.

Interpretation of these findings should be considered in light of study design and risk-of-bias assessments. Evidence supporting improvements in spatial understanding and planning accuracy is primarily derived from randomized controlled trials with low-to-moderate risk of bias. In contrast, findings related to workflow integration, implementation feasibility, and user acceptance are largely based on observational and feasibility studies, which demonstrated greater methodological heterogeneity and higher risk of bias. High-certainty conclusions were drawn where consistent RCT evidence existed (AR accuracy). Moderate-certainty conclusions arose from convergent randomized and observational evidence (VR spatial understanding, selected AR efficiency outcomes). Lower-certainty conclusions (plan modification) were derived mainly from observational studies. Clinically, AR is best supported for precision-critical intraoperative tasks, while VR offers value in preoperative decision support.

Mao et al. (61) provided an in-depth analysis of VR-based surgical training platforms and concluded that immersive VR environments enhanced technical skills, improved operative confidence, and were superior to traditional 2D or textbook-based training. Our findings strongly corroborate these observations in a clinical context. For instance, VR-based planning in our review improved accuracy in breast tumor localization, fracture fixation, and hepatobiliary surgery, while also prompting modifications in surgical plans in up to 52% of lung segmentectomies and 45.4% of TAVR procedures. This suggests that the educational benefits reported by Mao et al. effectively translate into practical surgical gains. However, unlike Mao's focus on novice and trainee performance, our study includes data from experienced surgeons, indicating that even high-level professionals benefit from immersive visualization tools.

Magalhães et al. (62) conducted a systematic review focused on MR applications in the operating room, reporting positive but still preliminary results regarding surgical navigation and intraoperative decision-making. Our study expands on these findings by providing more concrete outcome metrics, such as shortened procedure durations in thoracic interventions. Moreover, while Magalhães et al. emphasized technical feasibility and user satisfaction, we documented actual changes in surgical performance, including plan adaptations and measurable improvements in execution. This further reinforces the argument that MR, though less mature than VR or AR in terms of adoption, holds substantial promise in operative integration.

Queisner and Eisenträger (63) systematically reviewed the role of VR specifically in surgical planning and identified its ability to enhance mental imagery, spatial awareness, and communication between multidisciplinary teams. Our review provides direct validation of these insights with clinical evidence—showing that VR led to measurable plan modifications in a wide array of procedures, from skull base neurosurgery to robotic-assisted nephrectomy. While Queisner and Eisenträger acknowledged the current gap in standardized protocols for VR integration, our findings suggest that these tools are already being pragmatically adopted in diverse settings, albeit often as adjuncts to traditional planning methods. Additionally, our inclusion of RCTs and observational data brings quantitative strength to the qualitative insights raised in their review.

Tang et al. (64) conducted a broad systematic review on the applications of immersive technologies—including VR, AR, and MR—in medical practice and education. Their findings highlighted the extensive use of these tools in preclinical anatomy education, clinical skill acquisition, and surgical simulation. Notably, while Tang et al. reported promising learner engagement and knowledge retention, our review focused specifically on intraoperative and preoperative outcomes, thus expanding the conversation from educational environments into real-world surgical applications. Furthermore, unlike Tang's review which categorized outcomes by teaching content and student perception, our study prioritized objective surgical endpoints such as deviation reduction, procedural time, and plan modification rates. However, both reviews converge on one critical point: immersive technologies significantly enhance spatial cognition and understanding of complex anatomy—skills that are equally vital in surgical planning.

When compared with prior systematic reviews, our findings both corroborate and extend the current evidence base. Tang et al. and Mao et al. primarily emphasized the educational potential of VR, highlighting improvements in anatomical learning, operative confidence, and skill acquisition among trainees. While our review aligns with these conclusions, it advances the discussion by demonstrating that the same immersive advantages persist in clinical practice, improving preoperative planning and influencing intraoperative decisions even for experienced surgeons. Similarly, Queisner & Eisenträger described VR as a powerful facilitator of spatial cognition and interdisciplinary communication but noted a lack of standardized integration into surgical workflows. Our synthesis supports these insights while showing that, despite the absence of universal protocols, immersive platforms are already being deployed pragmatically with measurable effects on surgical outcomes, such as plan modifications and reduced operative deviations. Magalhães et al. highlighted MR as technically feasible but insufficiently validated; our findings substantiate their caution yet add concrete clinical evidence of MR reducing procedural time and needle adjustments in thoracic procedures. Collectively, these comparisons suggest that while earlier reviews predominantly framed immersive technologies as promising educational or experimental tools, our review provides clinical-level validation of their effectiveness, underscoring a shift from potential to demonstrable utility in modern surgical planning.

Beyond its applications in preoperative planning and intraoperative navigation, immersive technology has also demonstrated substantial promise in postoperative rehabilitation, effectively extending its impact across the surgical continuum. Virtual reality platforms have been shown to enhance physical recovery, pain management, and patient engagement following surgery. Naqvi et al. (65) emphasized the dual usability of VR for both patients and therapists, noting its ability to improve adherence and streamline rehabilitation protocols, which ultimately contributes to time efficiency in recovery. Similarly, Martín Pérez et al. (66), in their systematic review and meta-analysis of VR exposure therapy following cruciate ligament reconstruction, demonstrated superior functional outcomes and faster return of mobility compared with conventional rehabilitation, aligning closely with the precision and efficiency goals highlighted in our findings for surgical planning.

The benefits of VR also extend to pain control and patient-centered decision-making in postoperative care. Malik et al. (67) reported that VR serves as an effective adjunct for postoperative pain management, reducing opioid requirements and thereby supporting more informed clinical decision-making regarding analgesic strategies. Complementing this, Ehioghae et al. (68) found VR-based rehabilitation to improve functional recovery, range of motion, and patient-reported outcomes in orthopedic surgery, underscoring its role in achieving accurate and measurable postoperative improvements. Collectively, these findings suggest that immersive technologies not only optimize accuracy and efficiency during the surgical process but also sustain these benefits into the postoperative phase by accelerating recovery timelines and supporting evidence-driven therapeutic choices. As such, immersive platforms represent a comprehensive toolset capable of enhancing surgical care at every stage—from preoperative planning through intraoperative execution to postoperative rehabilitation.

The findings of this review highlight the emerging role of immersive and interactive technologies as tools for precision, efficiency, and adaptability in surgery. Rather than serving as competing modalities, VR, AR, and MR appear to function as complementary instruments across the surgical continuum: VR is particularly suited for preoperative planning and spatial rehearsal, AR enhances intraoperative navigation and precision, and MR provides real-time interactive integration of anatomical data. This multimodal approach reflects a paradigm shift toward digitally augmented surgery, where different technologies are tailored to specific tasks to collectively enhance surgical performance.

Evidence strength varied across outcome domains and study designs. Evidence supporting improvements in spatial understanding and planning accuracy was moderate, as these outcomes were frequently evaluated in randomized controlled trials with low-to-moderate risk of bias and showed relatively consistent findings. In contrast, evidence for time efficiency and workflow-related outcomes was low to moderate, as it was predominantly derived from observational and feasibility studies with greater methodological heterogeneity and higher risk of bias. Evidence regarding plan modification and decision-making changes was low, given reliance on small sample sizes, subjective outcome measures, and non-randomized designs.

Beyond their technical benefits, immersive technologies carry broader clinical implications. Improved visualization not only enhances surgical accuracy but also strengthens communication among team members, supports multidisciplinary collaboration, and facilitates patient education through more intuitive models of anatomy and surgical strategy (69). In the longer term, the affordability and scalability of these tools may help democratize access to advanced surgical planning, particularly in resource-limited settings. Nonetheless, the widespread adoption of these technologies will require addressing several challenges, including heterogeneity in study designs, lack of standardized outcome measures, and the need for robust cost-effectiveness data. These considerations provide a foundation for the future research directions discussed below.

## 5 Limitations

This review has a set of limitations that must be addressed. To start with, a formal meta-analysis was not conducted in this review due to substantial heterogeneity across the included studies. This heterogeneity primarily arose from variability in surgical procedures examined (ranging from orthopedic and neurosurgical to abdominal interventions), inconsistency in outcome definitions (for example, some studies assessed operative time while others focused on spatial accuracy, complication rates, or subjective surgeon confidence), and diversity in the application of immersive technologies (VR, AR, and MR were implemented in distinct ways and at different stages of the surgical workflow, such as preoperative planning, or intraoperative navigation). These methodological differences limited the feasibility of pooling results into a unified quantitative synthesis. Details of these heterogeneities are already captured in (Tables 1, 2). Moreover, although randomized controlled trials were part of the study, most of the evidence came from small-scale observational or feasibility studies which are likely to be more biased. Additionally, most studies focused on short-term outcomes with limited information pertaining to long-term clinical or patient-centered benefits. Most importantly, external validity is problematic given that most studies were conducted in high-resource settings and few investigated cost, setup complexity, and scalability. Lastly, multiple major databases were searched, the omission of Embase, Scopus, and the Cochrane Library may have resulted in the exclusion of some relevant studies, introducing potential retrieval bias.

## 6 Conclusion

This systematic review illustrates that immersive technologies are no longer experimental adjuncts but are emerging as practical tools for surgical precision, efficiency, and collaboration. Clinically, their integration into preoperative planning and intraoperative workflows could streamline surgical pathways, reduce complications, and enhance patient-centered care. Virtual Reality was particularly effective in enhancing anatomical understanding and prompting preoperative plan modifications, while Augmented Reality and Mixed Reality showed consistent improvements in procedural accuracy and intraoperative workflow. From a technological standpoint, future platforms may increasingly combine immersive visualization with AI, robotics, and real-time data analytics, enabling more adaptive and intelligent surgical environments. However, limitations of this review—including heterogeneity of study designs, lack of standardized outcomes, and the predominance of single-center studies—underscore the need for multicenter RCTs, harmonized reporting frameworks, and cost-effectiveness analyses. Future research should also investigate patient-level benefits, such as recovery times and satisfaction, and explore ways to democratize access to immersive technologies in low-resource healthcare systems.

## Data Availability

The original contributions presented in the study are included in the article/Supplementary Material, further inquiries can be directed to the corresponding author.
