# Supplementary material for: The digital evolution of surgical planning: a systematic review of immersive and interactive technologies
Source: Front Surg. 2026 Feb 26;13:1764132. doi: 10.3389/fsurg.2026.1764132 (PMC12979114; doi:10.3389/fsurg.2026.1764132)
Supplement: Supplementary file 1 [file Supplementaryfile1.docx]

**Supplementary Material**

**Appendix 1**

**Search Strategy**

| **Ovid MEDLINE** | | |
| --- | --- | --- |
| **#** | **Query** | **Results** |
| 1 | Mixed reality.ab,at. | 194 |
| 2 | Augmented reality.ab,at. | 767 |
| 3 | Virtual reality.ab,at. | 3,783 |
| 4 | Preoperative planning.ab,at. | 4,004 |
| 5 | Surgery.ab,at. | 531,530 |
| 6 | 1 or 2 or 3 | 4,507 |
| 7 | 4 or 5 | 533,958 |
| **8** | **6 and 7** | **988** |

| **PubMed** |
| --- |
| ("Mixed reality" OR "Augmented reality" OR "Virtual reality") AND ("Surgery" OR "Preoperative planning"). **Filters: RCTs only. Results: 568** |

| **Google Scholar** |
| --- |
| (“Mixed reality” OR “MR” OR “Augmented reality”) AND (“Surgery”). **Filters: Exact phrases. Results: 244** |

| **Web of Science** |
| --- |
| Mixed reality (Title) or Augmented reality (Title) or Virtual reality (Title) and Preoperative planning (Title) and **Surgery (Web of Science Categories) and Article (Document Types)** **Results: 574** |

**Appendix 2**

1. **Quality assessment results using RoB2 tool**

**
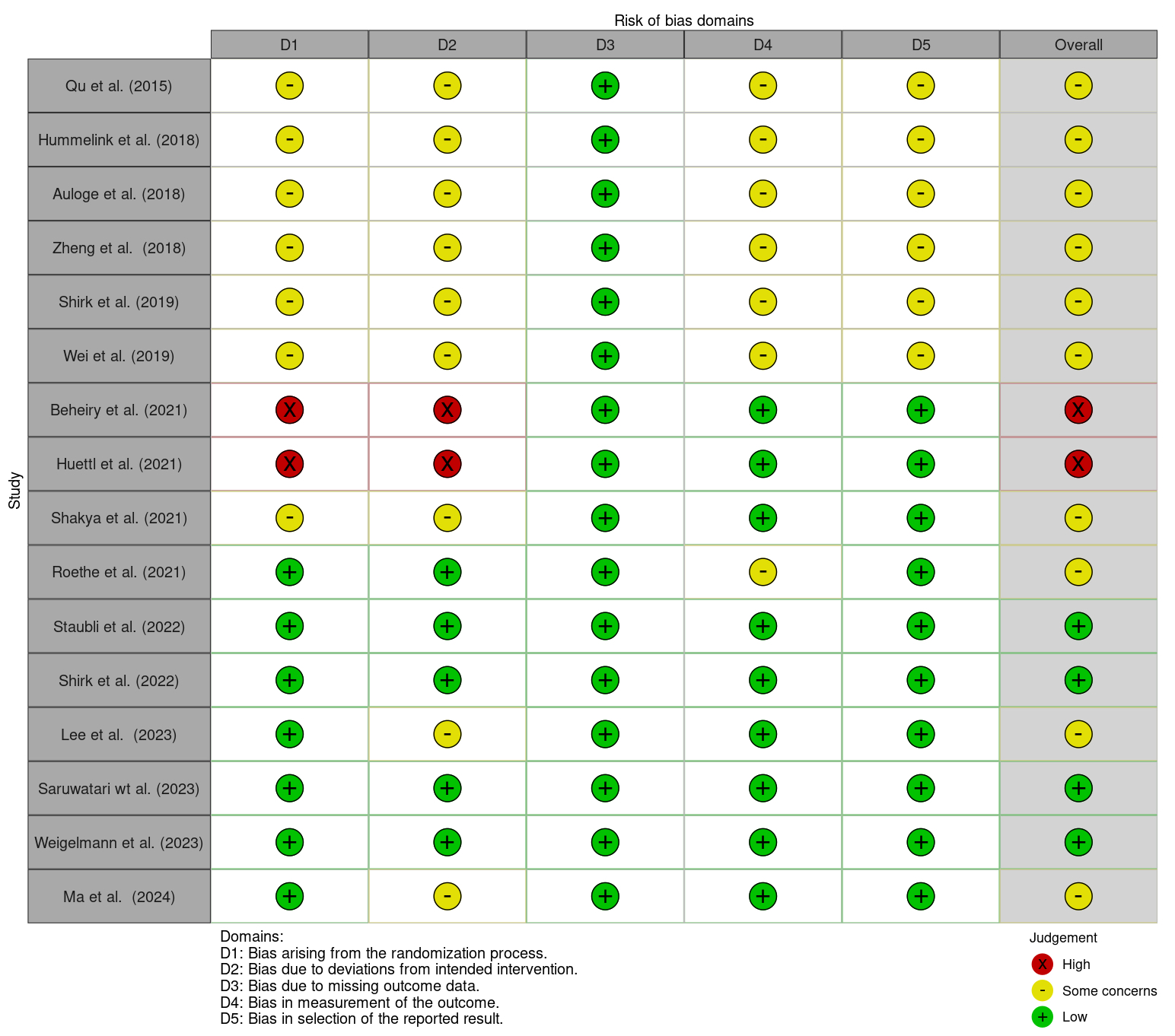
**

**
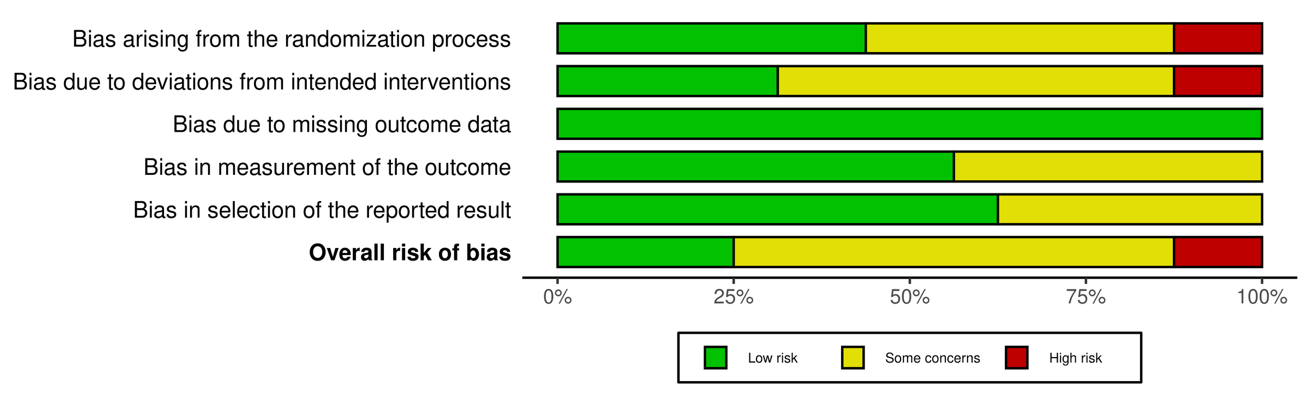
**

1. **
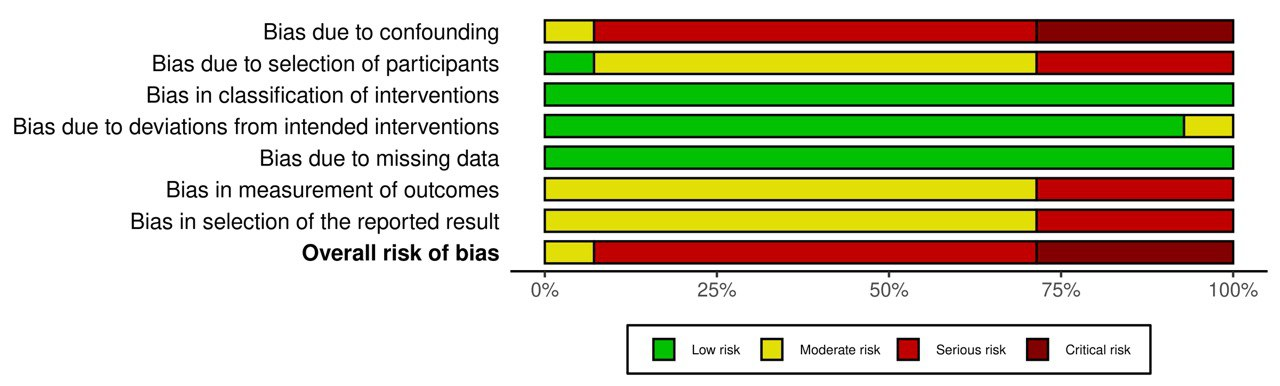

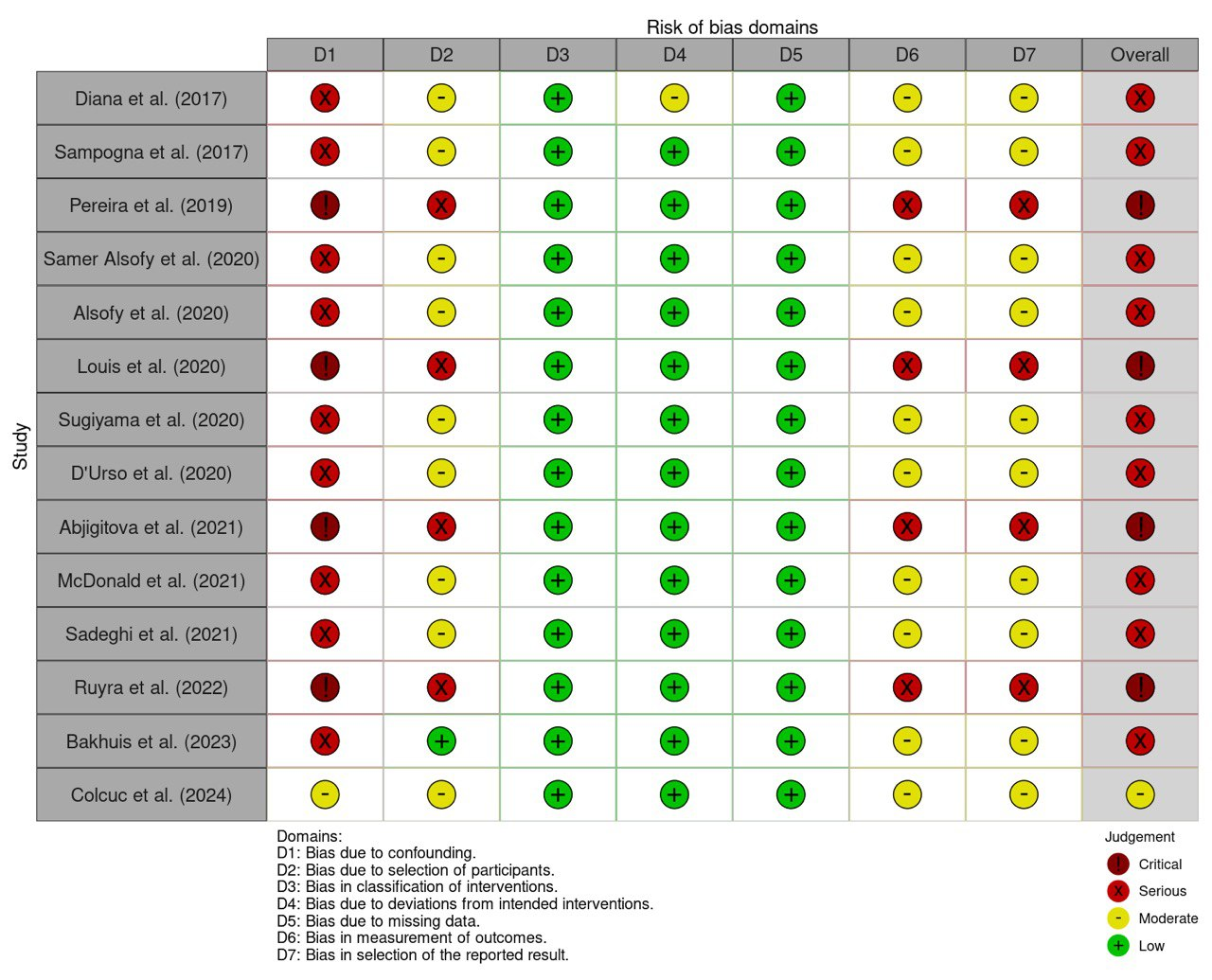
Quality assessment results using ROBINS-I tool**
